# Development of an Endoscope Reprocessor Using Ozonated Water Jets

**DOI:** 10.1007/s10439-026-04109-6

**Published:** 2026-04-16

**Authors:** Maycon C. O. Carvalho, Tatiana R. O. Heinzelmann, Leandro L. Azevedo, Adriana B. Fernandes, Carlos J. de Lima

**Affiliations:** 1 Department of Biomedical Engineering, Anhembi Morumbi University, São José dos Campos, SP Brazil; 2 Center for Innovation, Technology and Education (CITÉ), São José dos Campos, SP Brazil

**Keywords:** Biomedical equipment, Disinfection, Endoscope, Ozone, Reprocessing

## Abstract

**Objective:**

To develop an endoscope reprocessing system using ozonated water jets.

**Methods:**

The system features a jet head with concentric orifices that moves vertically to disinfect the external surface of the endoscope. Simultaneously, ozonated water is injected into the internal channel to ensure complete disinfection. Endoscopes were experimentally contaminated with *Staphylococcus aureus* and *Escherichia coli*. Quantitative and qualitative microbiological analyses were performed to evaluate the effectiveness of enzymatic detergent washing followed by ozonated water disinfection.

**Results:**

The system achieved uniform disinfection across all endoscope surfaces. Quantitative analysis demonstrated a 4-log (99.99%) reduction in colony-forming units (CFU/mL) on the endoscope and in the post-disinfection water. Qualitative analysis showed no turbidity, indicating the absence of bacterial growth.

**Conclusion:**

This preliminary study presents the development of an endoscope reprocessor capable of achieving a 99.99% reduction in bacterial load using ozonated water jets. The system may represent a more resource-efficient alternative to conventional methods, consuming only 8 liters of water per cycle through recirculation and re-ozonation. The combined mechanical action of the water jets and the antimicrobial properties of ozone resulted in effective disinfection under the experimental conditions, reducing the processing time to 15 minutes compared to conventional methods.

## Introduction

Endoscopes are indispensable tools, facilitating the diagnosis and treatment of multiple medical conditions [1]. Their applications range from gastrointestinal procedures to respiratory examinations, providing direct visualization of internal organs [2]. Due to their direct contact with mucous membranes and internal tissues, combined with their complex design and narrow lumens, endoscopes must be properly reprocessed to ensure microbiological safety [3, 4].

In Brazil, the reprocessing of medical devices is regulated by the National Health Surveillance Agency (ANVISA), primarily through RDC No. 156/2006 and RDC No. 6/2013. These regulations establish standards for the manufacturing, marketing, and reprocessing of critical devices such as endoscopes [5, 6]. They require validated disinfection protocols, product registration, traceability, compatibility studies between materials and chemical agents, and safe disposal protocols. Internationally, the U.S. Food and Drug Administration (FDA) and International Organization for Standardization (ISO) provide similar guidelines. The FDA recognizes standards such as ISO17664, which include rigorous testing and detailed instructions to ensure the effectiveness of cleaning, disinfection and sterilization processes [7, 8].

According to guidelines from World Gastroenterology Organization (WGO) and ANVISA, endoscope reprocessing involves several interdependent steps to ensure both patient and healthcare worker safety. These steps include pre-cleaning, transport, leak testing, cleaning, rinsing, disinfection, drying, and storage [1, 2].

However, the disinfection of endoscopes presents significant challenges due to the structural complexity of these devices and the nature of endoscopic procedures. One of the primary difficulties lies in eliminating pathogens that may lodge in hard-to-reach areas, such as internal channels and articulated components [9]. Moreover, the time and resources required for proper disinfection are considerable, particularly in clinical settings with high demand for endoscopic procedures. The choice of disinfecting agents must consider not only antimicrobial efficacy but also patient and healthcare worker safety, as well as environmental impact [10].

In response to these challenges, innovative approaches have been explored. Zveny et al. [11] highlighted the use of cold plasma as a promising alternative to conventional disinfection methods due to its ability to act effectively on complex surfaces. However, this technique caused minor structural alterations, such as increased surface roughness and decreased hydrophobicity, underscoring the need for technologies that combine efficacy and material preservation.

Ozonated water has demonstrated high efficacy in disinfecting various materials and biomaterials. Awoyama et al. [12] used a hydrodynamic system with ozonated water to disinfect human amniotic membranes and reported the absence of microbial growth after treatment. Similarly, Heinzelmann et al. [13] demonstrated the effectiveness of a hydrodynamic system combining ozonated water and ultrasound in removing bacteria from surgical instruments, achieve efficient disinfection.

More broadly, ozonated water has emerged as a viable alternative for endoscope disinfection due to its bactericidal and virucidal properties. When dissolved in water, ozone forms a potent oxidizing solution capable of eliminating pathogenic microorganisms [14, 15]. Marson et al. [16] demonstrated the use of a hydrostatic ozonated water system with a porous diffuser to disinfect gastroscopes. More recently, Carvalho et al. [17] developed a hydrodynamic device that submerges the colonoscope and injects ozonated water directly into the working channel, improving fluid circulation and enhancing the oxidative activity of ozone.

Disinfection of endoscopes can be performed manually or through automated system, each with specific characteristics that influence effectiveness and applicability. In manual disinfection, the endoscope undergoes physical cleaning and immersion in disinfectant solutions, with the operator controlling exposure time and chemical concentration [5]. While this method is more accessible and flexible, it relies heavily on operator skill, which can lead to errors and inconsistencies that compromise disinfection and increase contamination risk [18].

In contrast, automated disinfection employs specialized equipment that performs cleaning and disinfection through standardized and controlled cycles, including automated delivery of disinfectants and rinsings phases. This method enhances consistency, traceability, and reduces variability and the risk of cross-contamination [19].

Chemical agents such as peracetic acid, glutaraldehyde, and orthophthaldehyde (OPA) are widely used in both manual and automated processes. However, these compounds pose significant limitations. They have toxic potential, causing respiratory, ocular, and dermal irritation, and require strict safety measures. Additionally, they may be incompatible with certain endoscope materials, potentially causing equipment degradation. Their disposal also poses environmental challenges. Moreover, their effectiveness often depends on prolonged exposure times, and their use is associated with high operational costs. Effective and safe endoscope reprocessing requires a thorough understanding and management of these limitations [20–22].

Ozone (O_3_) offers several advantages over traditional chemical disinfectants due to its high efficacy against a broad spectrum of microorganisms. It acts rapidly, leaving no chemical residues because as it quickly decomposes into oxygen, thereby minimizing environmental impact and simplifying waste management [23]. Its material compatibility also reduces the risk of equipment damage. Additionally, on-site ozone generation eliminates the logistical costs associated with transport and storage, making it a safe, sustainable, and efficient alternative for disinfecting medical devices [23, 24]. Therefore, this study aimed to develop an endoscope reprocessor based on concentric jets of ozonated water.

## Methods

### Type of Research

This study is classified as experimental and applied research, focusing on the development and evaluation of the feasibility and effectiveness of an endoscope reprocessing system.

### System Development

**Fig. 1:**
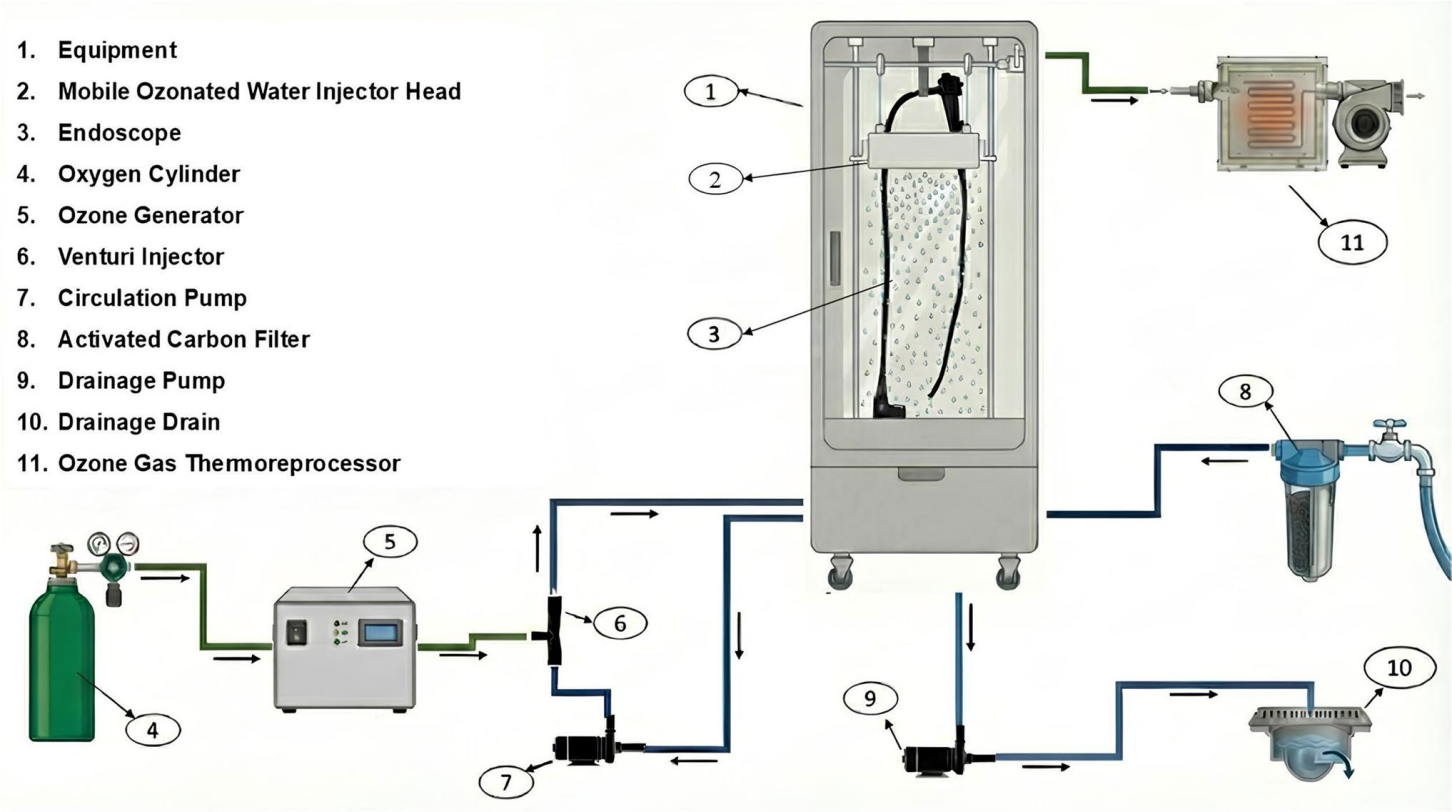
The figure illustrates the operational flow and key components of the process. Internal elements, such as pumps, the reservoir, and sensors, are housed within the equipment and have been omitted for visual clarity. *Source*: Authors

The ozonated water disinfection system (Fig. 1) consists of a main chamber equipped with two vertical guide rods that direct the movement of an injection head. The head contains 60 micro-orifices (0.5 mm each), inclined at 30°, producing concentric jets aimed at the center of the assembly. Vertical displacement of the head is driven by an electronically controlled motor, ensuring uniform application of ozonated water across the entire external surface of the endoscope.

Water is supplied to the injection head through two inlets connected to a self-priming pump operating at 760 kPa via silicone tubing. A second, independent hydraulic circuit, driven by a pump with similar specifications, delivers ozonated water directly to the endoscope’s working channel, ensuring effective internal disinfection.

Water drained from the external surfaces and internal channels is collected and returned to the main reservoir, where it is re-ozonated and recirculated, enabling the entire process to use approximately 8 liters of water per cycle. Replenishment with tap water is controlled by a solenoid valve and pre-treated via activated carbon filtration before entering the system.

Ozone is generated from medical-grade oxygen supplied to an MS3G generator (MS Ltd., Brazil), regulated at a flow rate of 0.25 L/min. The generator produces an O₂/O₃ mixture at a concentration of 45 mg/L, which is subsequently dissolved into the water via a Venturi injector and delivered to the reservoir. Prior to disinfection, the water undergoes a 25-minute pre-ozonation period, corresponding to the typical manual cleaning time recommended by international guidelines. During the subsequent 15-minute disinfection phase, continuous ozonation maintains a high concentration of dissolved O₃, which is monitored in real time using a DOZ3O sensor.

At the end of the cycle, effluent is safely removed through coordinated operation of the pump and drainage valve, allowing the ozonated water to be discharged into the public sewage system. The entire system is managed by a microcontroller, ensuring precise operation and standardized performance throughout the disinfection process.

## Experimental Contamination

Standardized strains of *Staphylococcus aureus* (CCCD S013) and *Escherichia coli* (CCCD E008) were cultured separately on Tryptic Soy Agar (TSA- KASVI®) and incubated at 37 °C for 24 hours. Bacterial suspensions were prepared in sterile 0.9% sodium chloride solution.

The McFarland nephelometric scale was used to standardize bacterial concentrations, measured using a turbidimeter (Plus, Alfakit), calibrated with ultrapure water and validated against reference standards. Final suspensions were adjusted to 10⁶ CFU/mL.

For external contamination, a flexible endoscope (Pentax, EC-383IL) was immersed in a disinfected glass container with 250 mL of the bacterial suspension and incubated for 30 minutes. For internal contamination, 3 mL of the same suspension was injected directly into the working channel using a sterile syringe.

## Disinfection Protocol

Disinfection procedures were performed in a temperature-controlled environment (20 °C). After contamination, the endoscope was rinsed with distilled water and immersed in an enzymatic detergent solution (Zymedet Gold, Prolink®) for 5 minutes, following the manufacturer´s instructions. It was then rinsed again with distilled water and placed in the reprocessor for ozonated water disinfection.

## Microbiological Analysis

### Quantitative Analysis

Samples were collected from three regions of the endoscope (proximal, medial, and distal), totalling 15 samples per region for each phase of the process (post- contamination, after enzymatic detergent washing, and after ozonated water disinfection). Additionally, water samples were collected from the working channel, the reservoir, and the collector.

Endoscope samples were collected using sterile swabs applied to the specified regions. Each swab was suspended in sterile saline and plated onto TSA using the plate spreading technique. Plates were incubated at 37 °C, and bacterial growth was assessed after 24 and 48 hours.

For water analysis, 0.1 mL of each sample was plated onto TSA using the same spreading method, with 15 samples per collection site. Bacterial growth was evaluated after 24 and 48 hours.

Data were expressed as mean ± standard deviation. Additionally, 95% confidence intervals (95% CI) were calculated using a t-distribution (n = 15) to provide a complementary estimate of data variability.

### Qualitative Analysis

For qualitative analysis, fragments of silicone and polytetrafluoroethylene (PTFE) materials commonly used in endoscope manufacturing were analyzed. After undergoing contamination, enzymatic washing, and ozonated water disinfection, 15 fragments of each material were immersed in sterile tubes containing Tryptic Soy Broth (TSB - KASVI®). Samples were incubated at 37 °C and visually assessed for turbidity after 24 and 48 hours. Turbidity was interpreted as indicative of bacterial viability.

## Hydrodynamic Parameters and Ozone Dosage

The hydrodynamic behavior of the system was characterized to understand the flow of the ozonated water flow during disinfection. The Reynolds number (Re) was calculated according to Equation 1 to determine the flow regime in the endoscope’s working channel, as the distinction between laminar and turbulent flow affects both the efficiency of organic matter removal and the distribution of ozonated water along the channel.

The kinetic energy of the jets was estimated based on the outlet velocity of water through the 60 micro-orifices (0.5 mm each) of the injection head. This parameter quantifies the mechanical impact of the microjets on the endoscope surface, as the energy transferred by the flow contributes to the disaggregation and removal of adhered organic matter. Thus, the kinetic energy analysis aids in characterizing the system’s hydrodynamic contribution to the cleaning process, complementing the oxidative action of dissolved ozone.

The applied ozone dosage (Equations 3 and 4) during the disinfection process was estimated based on the dissolved ozone concentration in water and the gas flow supplied to the generator. The calculation followed the method proposed by van Leeuwen [25] to quantify ozone dose in liquid media, considering the mass of ozone transferred to the system per unit time and its distribution throughout the total water volume used in the cycle. This methodology employed the ozone concentration produced by the generator (Cₒ₃), the oxygen feed flow rate, and the operational volume of the hydraulic reactor. This approach enables the determination of the total exposure of the endoscope to the oxidizing agent and facilitates standardized comparisons across different operational configurations.

## Physicochemical Parameters of the Water

Monitoring of the water’s physicochemical parameters was carried out to characterize the operational conditions during the pre-ozonation and disinfection stages. The water used, sourced from the municipal supply network, underwent activated carbon filtration (Equation), providing free-chlorine reduction and Class D particle retention. The pH was assessed using Macherey-Nagel indicator strips and a PG3000 digital pH meter (Gehaka), with measurements taken before and after each stage (pre-ozonation and disinfection). Temperature was continuously monitored using a Minipa MT-450A digital thermometer. These parameters allowed the characterization of the physicochemical stability of the solution during recirculation and ozonation.

## Results

**Fig. 2:**
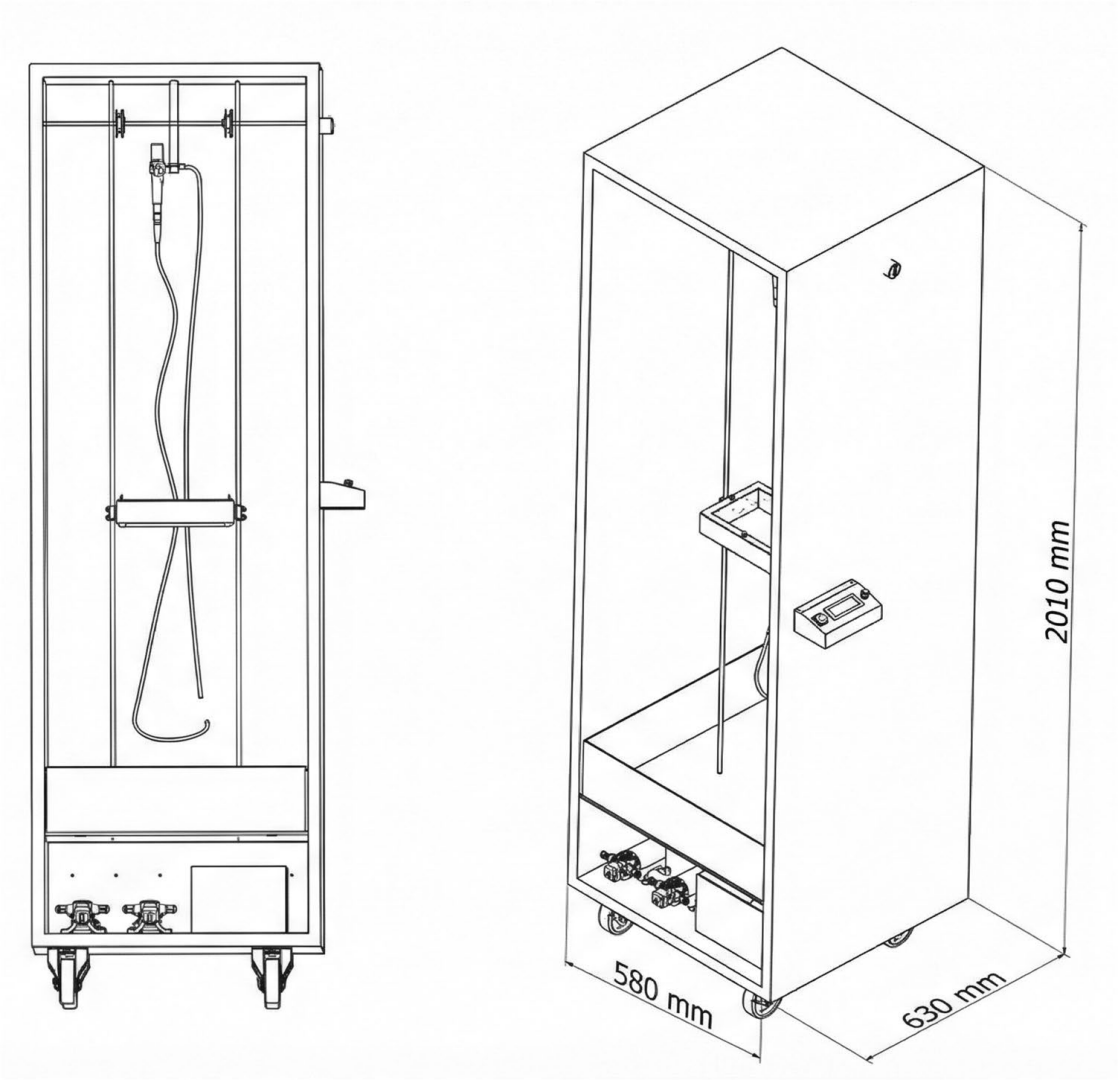
Three-dimensional illustration of the endoscope disinfection system, created using Autodesk Inventor^®^, providing an overview of the equipment

The endoscope reprocessing equipment (Fig. 2) employed an innovative system of concentric ozonated water jets, ensuring uniform coverage of all external surfaces of the endoscope, including hard-to-reach areas such as the internal channels.

The system continuously injected ozonated water into the endoscope's working channel, achieving a flow rate of 1.74 L.min⁻^1^ at the distal end. Considering the internal diameter of the working channel (2.8 mm), the average flow velocity was calculated as 4.71 m.s^-1^. The Reynolds number was determined using Equation (1):

1 $$\begin{gathered} Re = \frac{p . v . D}{\mu } \hfill \\ Re = \frac{{1000 Kg.m^{ - 3} . 4.71 m.s^{ - 1} . 2.8 \times 10^{ - 3} m}}{{1.003 \times 10^{ - 3 } Pa.s}} \hfill \\ Re = 13.147 \hfill \\ \end{gathered}$$

This result indicates a turbulent flow regime of ozonated water inside the PTFE pipeline.

The water pump operated at 760 kPa, delivering a flow rate of 3L.min^-1^ to the injector head, which contains 60 orifices. Each orifice projected a jet of ozonated water with individual kinetic energy calculated using Equation (2):

2 $$\begin{gathered} E_{c} = \frac{1}{2}mv^{2} \hfill \\ E_{c} = \frac{1}{2}.8.333 \times 10^{ - 4} Kg.\left( {4.245m.s^{ - 1} } \right)^{2} \hfill \\ E_{c} = 7.5 \times 10^{ - 3} J \hfill \\ E_{total} = 60 . 7.5 \times 10^{ - 3} J = 0.45 J. \hfill \\ \end{gathered}$$

**Fig. 3:**
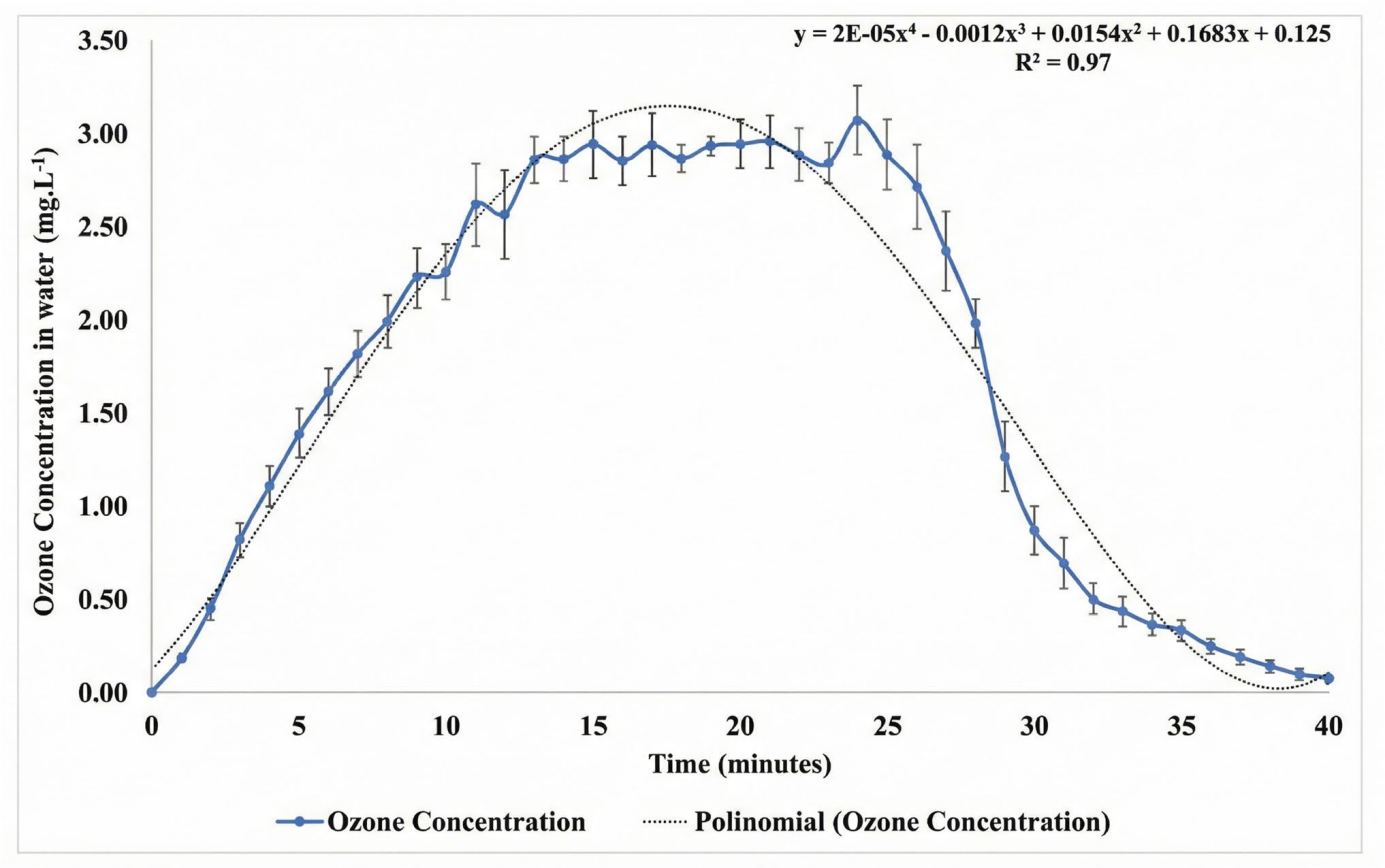
Dissolved ozone concentration profile during pre-ozonation and disinfection phases over time

Figure 3 shows the profile of dissolved ozone concentration in water during the 25-minute pre-ozonation and the subsequent 15-minute disinfection phases. The graph plots ozone concentration (y-axis) versus time (x-axis), illustrating the increase in concentration during pre-ozonation and the expected decrease after endoscope insertion due to ozone consumption. A polynomial trendline, along with its coefficient of determination (R^2^), is included to highlight the curve fitting and overall ozonation behavior throughout the cycle.

The total surface area of the endoscope exposed to ozone was 370 cm^2^, including 282 cm^2^ of external surface and 88 cm^2^ from the internal working channel. The ozone generator, supplied with O₂ at a flow rate of 0.25 L·min⁻^1^, produced an ozone concentration (Cₒ₃) of $0.045 g.{L}^{-1}$, resulting in ozone feed rate, as shown in Equation (3). This calculation follows the approach proposed by van Leeuwen for quantifying applied ozone dosage in liquid media [25].

3 $$\begin{aligned} m_{O3} & = Q.C_{O3} = 0.25 L.\min^{ - 1} .0.045g.L^{ - 1} \\ & \, = 11.25 \times 10^{ - 3} g.\min^{ - 1} \\ \end{aligned}$$

The applied ozone dosage was calculated according to van Leeuwen [25], as shown in Equation (4):

4 $$\begin{aligned} Dosage & = \frac{{m_{O3} }}{{{ ^{\prime}A}{\mathrm{rea}}}} \\ Dosage & = \frac{{11,25 . 10^{ - 3} g.min^{ - 1} .15 min}}{{370cm^{2} }} \\ & \, = 4.56 \times 10^{ - 4} g.cm^{ - 2} \\ \end{aligned}$$

The water maintained an average temperature of 20 °C throughout the process, with no significant variations between the pre-ozonation and disinfection stages. Likewise, the pH remained at an average of 6.5, with no changes after ozone exposure, indicating physicochemical stability of the medium throughout the operational cycles.

Table 1 presents the quantitative microbiological analysis results for four regions of the endoscope (proximal, medial, distal, and working channel). The calculated 95% confidence intervals were narrow across all regions, reflecting low variability and good reproducibility of the experimental results.

**Table 1:** Quantitative microbiological analysis (CFU/mL) of endoscope regions

Regions	Control	ED	O_3_
*S. aureus* (CFU/mL)			
Proximal	5.8 × 10^4^ (±0.01)	2.4 × 10^3^ (±0.02)	–
Medial	6.8 × 10^4^ (±0.02)	3.8 × 10^3^ (±0.06)	–
Distal	6.2 × 10^4^ (±0.02)	3.7 × 10^3^ (±0.06)	–
Working channel	8.8 × 10^4^ (±0.08)	7.2 × 10^2^ (±0.01)	–
*E. coli* (CFU/mL)			
Proximal	6.7 × 10^4^ (±0.02)	2.2 × 10^3^ (±0.01)	–
Medial	7.1 × 10^4^ (±0.03)	4.1 × 10^3^ (±0.01)	–
Distal	7.5 × 10^4^ (±0.05)	4.8 × 10^3^ (±0.07)	–
Working channel	8.1 × 10^4^ (±0.04)	1.0 × 10^3^ (±0.01)	–

–indicates absence of bacterial growth; ED, Enzimatic Detergent

**Fig. 4:**
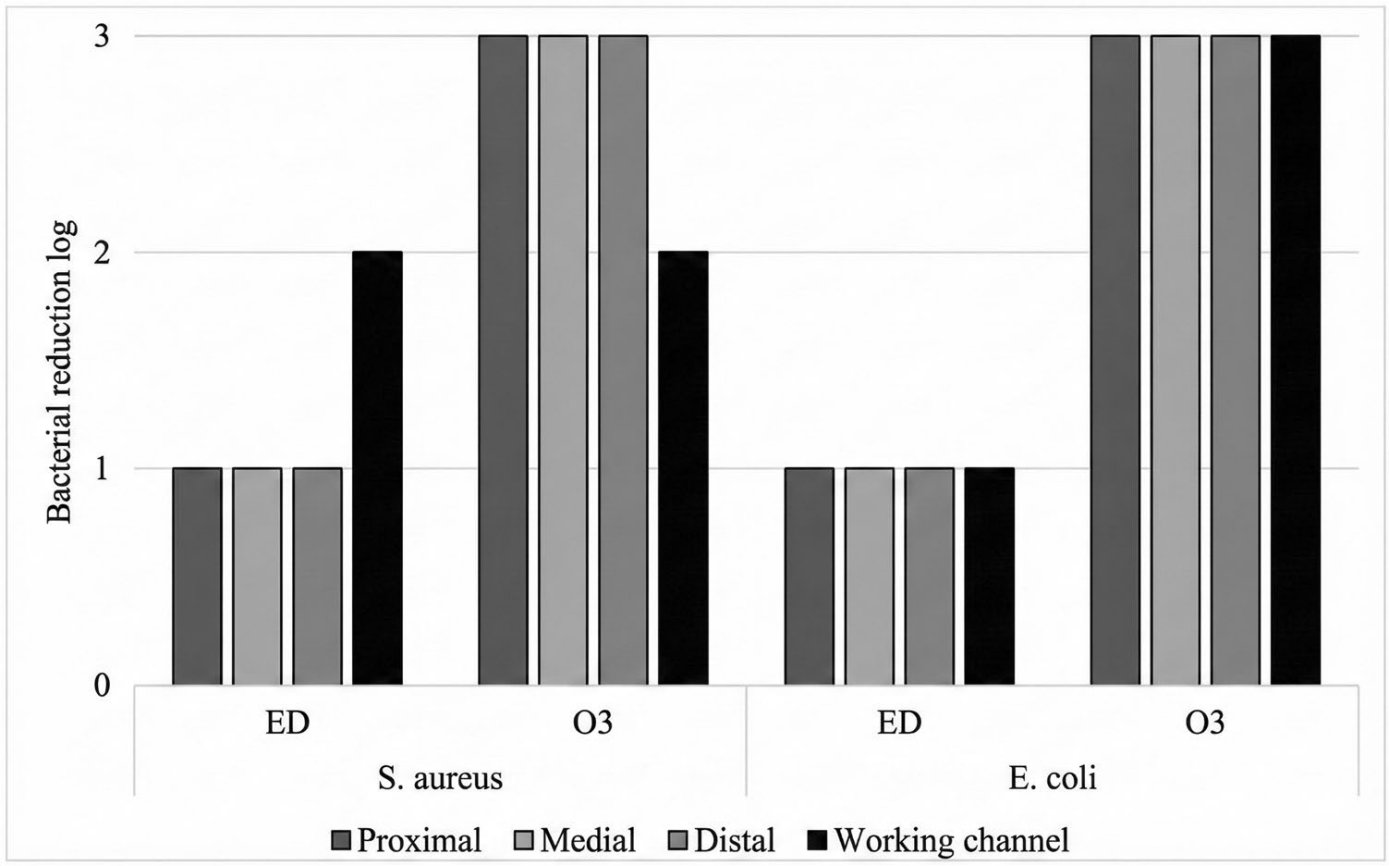
Logarithmic reduction in CFU/mL across endoscope regions following disinfection

Figure 4 illustrates the logarithmic reduction in CFU/mL across different endoscope regions after enzymatic detergent washing and subsequent ozonated water disinfection. The complete reprocessing protocol achieved a 4-log reduction, equivalent to a 99.99% decrease in bacterial load.

It is also worth noting that microbiological analysis of water samples collected from the collector and reservoir after the disinfection process revealed no bacterial growth, confirming the effectiveness of the system.

The qualitative microbiological analysis of silicone and PTFE fragments is summarized in Table 2. All fragments showed turbidity after contamination and enzymatic detergent washing. However, no turbidity was observed after ozonated water disinfection, indicating the absence of bacterial growth.

**Table 2:** Qualitative microbiological analysis of silicone and PTFE fragments

Group	*S. aureus*		*E. coli*	
	Silicone	PTFE	Silicone	PTFE
Positive control	+	+	+	+
Negative control	–	–	–	–
ED	+	+	+	+
O_3_	–	–	–	–

ED, Enzimatic Detergent; “ + ” = turbidity (growth); “–” = no turbidity (no growth).

## Discussion

The device developed in the present study demonstrated effective endoscope disinfection through the application of concentric jets of ozonated water, providing both mechanical (kinetic) and oxidative actions. The kinetic energy of the water jets (with a total kinetic energy delivered every second) facilitated the removal of organic residues from the endoscope surfaces, while ozonated water acted to eliminate microorganisms. These findings align with those of Carvalho *et al.* [17], who also reported effective disinfection using a hydrodynamic ozonated water system.

Previous studies indicate that ozonated water presents significant advantages over traditional chemical disinfectants, reducing toxic waste and minimizing occupational hazards [13, 16, 26]. The concentration and exposure time of ozone are critical for ensuring microbiological efficacy, as demonstrated by Urata *et al.* [26] in their study on automated endoscope disinfection. Moreover, integrating automated systems with standardized reprocessing protocols can enhance reproducibility and reduce the variability associated with manual disinfection [7].

In the disinfection of the endoscope channel, the hydrodynamic action of ozonated water resulted in a Reynolds number of 13.147, indicating turbulent flow within the PTFE tubing. This flow regime promotes cavitation, which hinders microbial adhesion and colonization [27, 28].

The pre-ozonation time was set at 25 minutes, corresponding to the average duration of initial cleaning steps, including pre-washing, leak testing, manual washing, enzymatic detergent application, and rinsing [29]. According to Iwakiri *et al.* [15], these initial steps are critical to reduce organic matter, which can react with ozone and decrease its concentration and disinfectant activity. During these preliminary steps, water is pre-ozonated. The endoscope is then placed into the system, where it remains for 15 minutes. This duration was sufficient to achieve bacterial elimination, completing the disinfection process in less time than the conventional method, which typically lasts about 30 minutes [30].

Figure 3 illustrates the ozone concentration in water during the disinfection stage, showing a steady increase during pre-ozonation and a peak of 3.07 mg·L⁻^1^. A subsequent decrease was observed upon endoscope insertion, attributed to ozone interaction with residual organic matter [14, 15].

In the present study, *Staphylococcus aureus* and *Escherichia coli* were selected as contaminants due to their prevalence in the gastrointestinal tract [31] and their frequent detection on endoscopes [20, 32]. Quantitative microbiological analysis revealed that ozonated water jets effectively eliminated *S. aureus* and *E. coli* from proximal, medial, and distal surfaces, as well as from the working channel, achieving consistent bacterial reductions of 2 to 3 logs.

Compared with Marson *et al.* [16], who used a hydrostatic ozonated water system at 4 mg/L for 15 minutes, the developed system achieved equal or superior bacterial reduction despite a initial bacterial load approximately four times higher, reinforcing the efficacy of ozonated water jets in high-contamination scenarios.

Quantitative microbiological data indicated that reprocessing with ozonated water achieved a 4-log bacterial reduction, corresponding to 99.99% efficacy. This outcome surpasses that reported by Marson *et al.* [16], who achieved only a 2-log reduction using a hydrostatic system with longer exposure time. Furthermore, the absence bacterial growth in samples analyzed after 24 and 48 hours reinforces the reliability of the method and its capacity to ensure safe reprocessing.

Carvalho *et al.* [17] evaluated the efficacy of a hydrodynamic ozonated water system in reprocessing colonoscopes and demonstrated complete microbial elimination following disinfection. These findings are consistent with those of the present study, reinforcing the effectiveness of ozonated water in disinfecting, even the complex internal surfaces of endoscopes. Thus, the technology developed in this study represents a viable and innovative alternative to conventional chemical methods, offering a combination of microbiological efficacy, material compatibility, and environmental sustainability.

In addition to microbiological efficacy, the use of ozonated water reduces the need for conventional chemical disinfectants, such as peracetic acid and glutaraldehyde, while simplifying waste management. Although effective, these agents pose occupational risks and require prolonged exposure times [30, 33]. Ozone, in contrast, is a powerful oxidant that naturally decomposes into oxygen, eliminating the need for hazardous waste disposal. Moreover, the developed system uses only 8 liters of water per reprocessing cycle, whereas conventional methods that require large volumes and generate potentially harmful waste.

Material compatibility with disinfectants is crucial for the durability and safety of medical devices. Silicone and PTFE are commonly used in endoscope manufacturing due to their flexibility, thermal resistance, and low friction coefficient [11, 34]. According to the Madrid Declaration on Ozone Therapy [35], both materials exhibit excellent compatibility with ozone and are recommended for devices exposed to this oxidizing agent.

Qualitative microbiological analysis of silicone and PTFE fragments confirmed the effectiveness of ozonated water in eliminating microorganisms, as shown in Table 2. These findings align with previous studies demonstrating ozone´s potential to inactivate pathogens on hospital surfaces [36, 37]. These studies also highlighted ozone’s broad-spectrum antimicrobial activity, reinforcing its applicability in the disinfection of medical devices. Thus, the present findings suggest that ozonated water not only preserves the integrity of medical materials but also provides an effective and safe alternative to conventional chemical disinfection, ensuring microbiological safety of reprocessed equipment.

The results demonstrated the consistent performance of the system, although certain methodological considerations should be noted to guide future investigations. This study provided a reliable assessment of the system’s efficacy under controlled and standardized laboratory conditions. Future studies could include additional microorganisms and evaluate performance over multiple cycles, thereby strengthening the robustness of the findings and broadening the potential applications.

Beyond the experimental aspects, the results also provide insights into technology’s practical applications and clinical impact. The observed performance indicates that the system could support clinical workflows by providing standardized disinfection, reducing manual intervention, and minimizing chemical waste. The combination of hydrodynamic action and continuous ozonation enhances reproducibility and safety, making the system suitable for high-demand healthcare settings.

Based on these findings, several directions can be outlined to advance the technology’s development. Upcoming steps include clinical validation in hospital settings, assessment of performance over repeated cycles, expansion of the microbiological spectrum, and integration of additional sensors. These efforts will contribute to establishing the system as a scalable and technologically mature solution.

Overall, the findings indicate that the combination of hydrodynamic action and continuous ozonation constitutes an effective strategy for endoscope disinfection, providing microbiological efficacy, material compatibility, and potential clinical application. The system offers a promising alternative to conventional methods, meeting contemporary demands for safety, efficiency, and sustainability.

## Conclusion

This study describes the development and evaluation of an endoscope reprocessing system that performs disinfection through ozonated water jets, ensuring uniform contact with both external and internal surfaces of the device.

The system demonstrated high microbial efficacy, achieving significant reductions of *Staphylococcus aureus* and *Escherichia coli* by combining hydrodynamic action with the oxidative potential of ozone. In this context, it was observed that, in addition to the hydrodynamic action of the water jets, a portion of the flow drains by gravity along the longitudinal surface of the endoscope, providing additional support for the removal of residual organic material. In addition to its effectiveness, the system is environmentally sustainable, consuming only 8 liters of water per cycle, through recirculation and re-ozonation.

These results highlight the system's potential as a promising alternative to conventional endoscope disinfection methods. Future studies should focus on clinical validation and expansion of the microbiological scope to consolidate its applicability across different reprocessing scenarios.
